# Thorium-Based Iron Arsenide with Fe–Fe Bonding

**DOI:** 10.1021/acs.inorgchem.5c02207

**Published:** 2025-10-08

**Authors:** Nazar Zaremba, Mitja Krnel, Konstantin Semeniuk, Yurii Prots, Lev Akselrud, Ulrich Burkhardt, Andreas Leithe-Jasper, Eteri Svanidze, Yuri Grin

**Affiliations:** † Max Planck Institute for Chemical Physics of Solids, 01187 Dresden, Germany; ‡ Department of Inorganic Chemistry, 28270 Ivan Franko National University of Lviv, 79000 Lviv, Ukraine

## Abstract

This work presents a study on a new thorium iron arsenide, Th_2_Fe_12_As_7_, a second discovered compound in the ternary system. It crystallizes in the noncentrosymmetric hexagonal Zr_2_Fe_12_P_7_ structure type (space group *P* 6, *a* = 9.5506(4) Å and *c* = 3.8645(2) Å). The bonding analysis of Th_2_Fe_12_As_7_ reveals that the structure can be represented by a trigonal prismatic environment of the As atoms, which are the geometric locations of anionic components of the structure. The cationic components of the structure are represented by the Th species. Furthermore, the bonding analysis revealed a new feature—a three-dimensional framework of two-atomic bonds between the Fe atoms, which—in the sense of effective charges—is essentially neutral. It plays the role of a bonding mediator within the [Fe–As] framework. Th_2_Fe_12_As_7_ is a metallic paramagnet down to the lowest measured temperature, 0.4 K.

## Graphical abstract

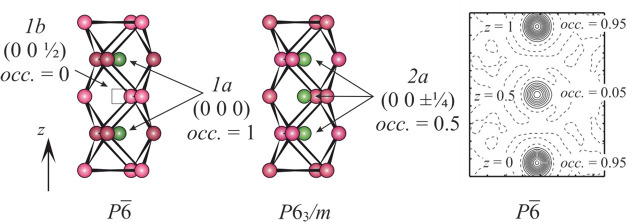

## I Introduction

Thorium is chemically similar to uranium, cerium, and several other lanthanides and actinides in terms of size, valence, and chemical bonding.^1,2^ When it comes to superconductivity, thorium-based compounds^3–11^ are somewhat more common than the uranium ones^3,12^ —they also tend to display higher critical temperatures, with the current record of 30 K for ambient (ThFeAsN)^6,13,14^ and 161 K for high pressure conditions (ThH_10_).^8,10^ To be fair, the majority of thorium-based superconductors are conventional in nature, while this is clearly not the case for the uranium-based systems, where unconventional superconductivity is more common. Among magnetic materials, thorium-based, unlike uranium-based compounds, are simpler since the actinide atom itself does not carry a magnetic moment. This means that in order to have magnetic ordering, thorium-based compounds should be combined with magnetic elements and are, in general, thorium-poor. A representative example of this is Th_2_Fe_17_, which orders ferromagnetically at 295 K.^15–17^ But the presence of magnetic elements does not guarantee that the compound will order magnetically, as we show by the example of Th_2_Fe_12_As_7_, which is the topic of this paper. Th_2_Fe_12_As_7_ does not show magnetic order down to the lowest measured temperature of 0.4 K. As we outline below, the nonmagnetic ground states can be understood from the point of view of chemical bonding: a 3D network of Fe–Fe bonds is virtually charge-neutral.

## II Materials and Methods

All sample preparation and handling were performed in the specialized laboratory,^18^ equipped with an argon-filled glovebox system (MBraun, p­(H_2_O/O_2_) < 0.1 ppm). Single crystals of Th_2_Fe_12_As_7_ were grown from the bismuth flux. Pure elements of Th (wire, Goodfellow, 99.98%), Fe (powder, ChemPur, 99.9%), As (pieces, Puratronic, 99.999%), and Bi (granules, ChemPur, 99.999%) in the ratio 1:4:2:20 were placed in an alumina Canfield-Svanidze crucible set^19^ and subsequently sealed in a Nb tube. Two methods were used for the synthesis. At the first attempt, the tube was heated to 1000 °C for 36 h, held at this temperature for 4 h, and slowly cooled to 700 °C for 196 h with further cooling to room temperature for 12 h in a vertical furnace. At the second attempt, the temperature was raised to 1200 °C, while the rest remained unchanged. In both attempts, the niobium tubes with the mixture were then sealed in a silica tube, heated to 500 °C, and placed into a centrifuge to remove bismuth flux from the sample. The first synthesis approach was unsuccessful; no signs of crystals of the title compound were detected. After the second try, a small yield of the shiny gray needle-shaped crystals of Th_2_Fe_12_As_7_ was recognized. Typically, crystals of this composition are grown along [001] from chunks of solid bismuth. Crystals appear to be stable in the air. As a byproduct, binary phases Th_3_As_4_ and Fe_2_As with elemental bismuth as a flux source, were observed. Powder X-ray diffraction was performed with a Huber G670 image plate Guinier camera with a Ge-monochromator (Cu Kα_1_ radiation, λ = 1.54056 Å). Phase identification was done using the Match 3! software.^20^ For the single-crystal experiment, specimens in the form of relatively thin (∼20–35 μm) elongated needles were used. The diffraction data were collected by using a Rigaku AFC7 diffractometer equipped with a Saturn 724+ CCD detector. The data reduction was performed by the Crystal Clear-SM Expert 2.0 package.^21^ Empirical absorption correction was performed by a multiscan routine.^22^ The WinCSD software package^23^ was used for structure solution, refinement, and data analysis. The complete crystallographic information and atomic coordinates with equivalent displacement parameters, together with interatomic distances, can be found in Tables 1, 2, S1, and S2, respectively.

The single crystals of Th_2_Fe_12_As_7_ were additionally analyzed by energy-dispersive X-ray spectroscopy with a Jeol JSM 6610 scanning electron microscope equipped with an UltraDry energy-dispersive X-ray spectroscopy (EDS) detector (Thermo Fisher NSS7). The semiquantitative analysis was performed with a 30 keV acceleration voltage. No impurity elements heavier than sodium were observed, confirming that no reaction with the crucible occurred during synthesis. The experimentally determined element ratio of Th:Fe:As was 9.4(2):56.6(2):34(2), which is in good agreement with the 9.5:57.2:33.3 composition obtained from structure refinement.

It was not possible to perform magnetic measurements or the analysis of specific heat since the Th_2_Fe_12_As_7_ phase was the minority one, comprising only 10% of the total sample. The accompanying impurities were Th_3_As_4_, Fe_2_As (antiferromagnetic below 350 K), and elemental Bi. Magnetic susceptibility measurements did not reveal any transitions that could not be attributed to impurities, suggesting that Th_2_Fe_12_As_7_ is paramagnetic. Additionally, a microscale device was prepared out of a Th_2_Fe_12_As_7_ single crystal using a plasma focused ion beam (FIB).^24^ The details of the procedure of micro device preparation are described elsewhere.^24–26^ AC electrical resistivity measurements were performed on a QD PPMS, using a standard four-probe technique at temperatures between *T* = 0.4 and 300 K in an *H* = 0 magnetic field. A current pulse of 0.01 mA with a frequency of 93 Hz for 1 s was applied along the [001] direction.

Calculations of electronic structure and analysis of chemical bonding were performed with the all-electron, local orbital full-potential method (FPLO^27^ (local density approximation as Perdew–Wang parametrization^28^) spin-polarized scalar relativistic calculation, standard basis set, 12 × 12 × 12 k points). The experimental lattice parameter and atomic coordinates obtained from crystal structure refinement in the space group *P* 6 and As1 in the origin were used for calculations within the LDA approach.

For the analysis of chemical bonding in position space, the electron-localizability approach was utilized.^29^ The electron density (ED) and the electron-localizability indicator (ELI-D) were calculated with a specialized module implemented in the FPLO program package.^30^ The topological features of the computed distributions of ED and ELI-D were analyzed with the program DGrid.^31^ To obtain information about the interacting atoms, the electron density was first integrated within atomic basins, i.e., spatial regions confined by zero-flux surfaces in the gradient field of ED. This technique represents the procedure proposed in the Quantum Theory of Atoms in Molecules (QTAIM)^32^ and provides effective electron populations for the QTAIM atoms. Further information about the bonding between atoms is obtained from the electron-localizability approach, combining the analysis of ED and ELI-D.^29^

## III Results and Discussion

The crystal structure of the Th_2_Fe_12_As_7_ compound is shown in Figure 1. The lattice parameters (Table 1) and element ratios of the new ternary arsenide of thorium suggest its crystal structure ascription to the structure type of Zr_2_Fe_12_P_7_.^33^

In the literature, the compounds of the 2:12:7 family are described with two models, a noncentrosymmetric in the *P* 6 space group^33,34^ and a centrosymmetric one in the *P*6_3_/*m* space group.^35,36^ The difference is mainly in the occupation of the positions on the [001] axis by the *p*-element (Figure 2 left, middle).

**Table 1:** Crystallographic Data of Th_2_Fe_12_As_7_ Refined with Two Models, Noncentrosymmetric and Centrosymmetric^a^

composition	Th_2_Fe_12_As_7_	
structure type	Zr_2_Fe_12_P_7_	Cl_2_F_12_Ba_7_
space group	*P* 6	*P*6_3_/*m*
*Z*	1	
Pearson symbol	*hP*21	
lattice parameters		
*a*, Å	9.5506(4)	
*c*, Å	3.8645(2)	
*V*, Å^3^	305.27(3)	
calcd density, g cm^–1^	9.023	
radiation	Mo Kα, λ = 0.71073 Å	
2θ range, deg	8.6–81.8	
range in *h*, *k*, *l*	–17 ≤ *h* ≤ 17	
	–17 ≤ *k* ≤ 14	
	–7 ≤ *l* ≤ 3	
absorption coeff., mm^–1^	57.164	
absorption correction	multiscan	
*T*(max)/*T*(min)	1.75	
*N*(*hkl*) measured	6187	
*N*(*hkl*) unique	1466	737
*R*(int)	0.0415	0.0508
*N*(*hkl*) observed	1412	728
observation criterion	*F*(*hkl*) ≥ 4σ [*F*(*hkl*)]	
refined parameters	49	31
*R*_F_	0.0391	0.0778
*R*_W_	0.0418	0.0848
residual peaks, e Å^–3^	1.61/–1.34	4.32/–2.68

a The cif file for the model in the space group *P* 6 has been deposited at the Cambridge Crystallographic Data Centre (CCDC number 2458374) and contains the supplementary crystallographic data for this paper. These data can be obtained free of charge via https://www.ccdc.cam.ac.uk/data_request/cif, by emailing data_request@ccdc.cam.ac.uk, or by contacting the Cambridge Crystallographic Data Centre, 12 Union Road, Cambridge CB2 1EZ, UK; fax: + 44 1223 336033.

**Table 2:** Atomic Coordinates and Equivalent Displacement Parameters (in Å^2^) of Th_2_Fe_12_As_7_ Refined in the *P* 6 Space Group^a^

atom	Wyckoff site	*x*/*a*	*y*/*b*	*z*/*c*	*U*_eq/iso_ (Å^2^)
Th1	1*d*	*^1^/_3_*	*^2^/_3_*	*^1^/_2_*	0.0095(2)
Th2	1*e*	*^2^/_3_*	*^1^/_3_*	0	0.0088(2)
Fe1	3*j*	0.3771(3)	0.9455(3)	0	0.0108(7)
Fe2	3*j*	0.2792(3)	0.1539(2)	0	0.0108(5)
Fe3	3*k*	0.6224(3)	0.0504(3)	*^1^/_2_*	0.0091(6)
Fe4^b^	3*k*	0.1004(3)	0.2149(3)	*^1^/_2_*	0.0119(6)
Fe5^c^	3*k*	0.122(6)	0.267(6)	*^1^/_2_*	0.012(6)
As1^b^	1*a*	0	0	0	0.0087(4)
As2	3*k*	0.4037(2)	0.1091(2)	*^1^/_2_*	0.0095(5)
As3	3*j*	0.5940(2)	0.8855(2)	0	0.0088(4)
As4^c^	1*b*	0	0	*^1^/_2_*	0.009(3)

a Atomic coordinates for the model refined in the *P*6_3_/*m* space group are presented in Table S1.

b Occupancy of Fe4 and As1 atoms are 0.95(1).

c Occupancy of Fe5 and As4 atoms are 0.05(1).

**Figure 1:**
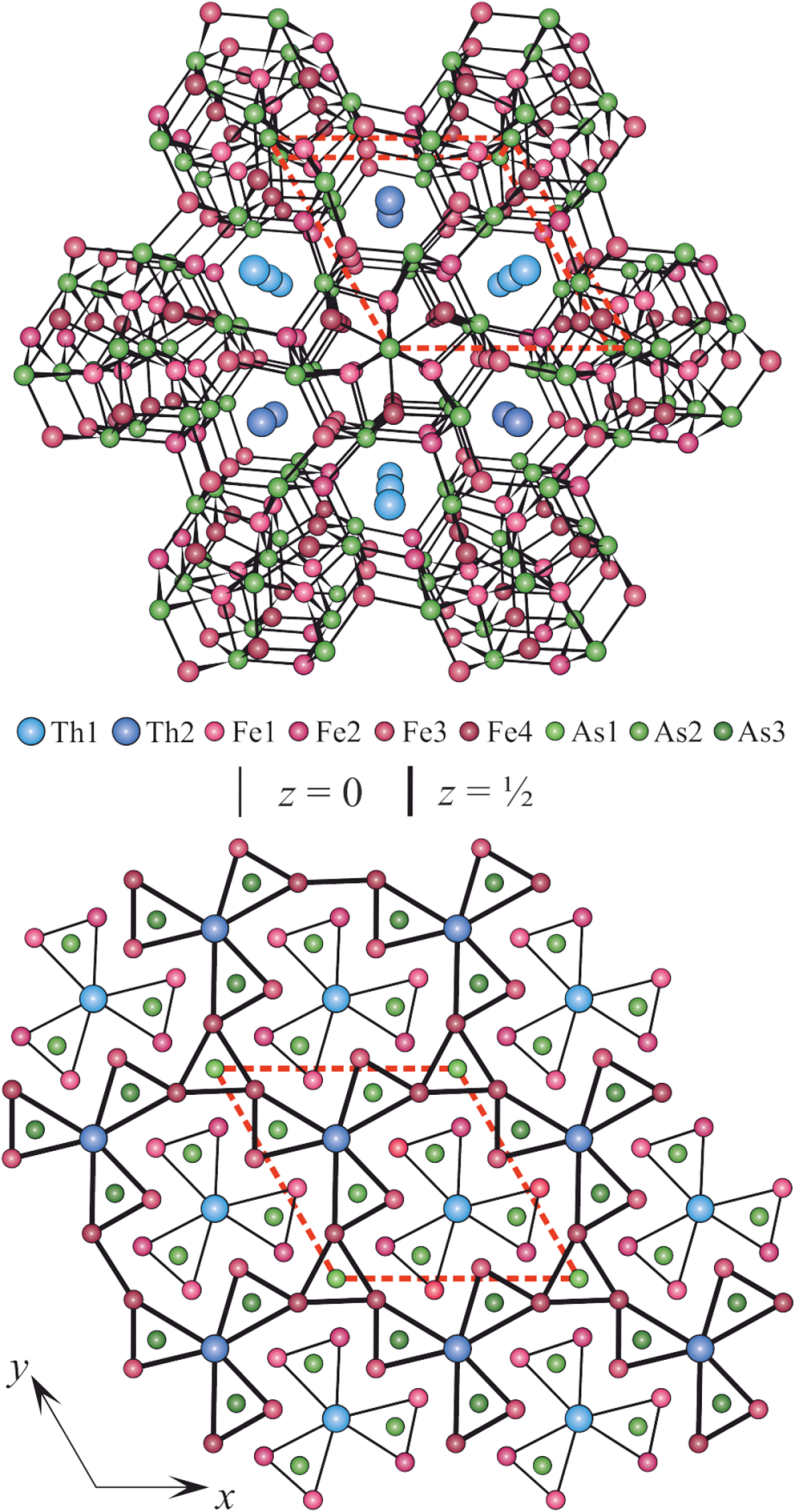
Crystal structure of Th_2_Fe_12_As_7_ represented as a network of interconnected Fe and As atoms (top panel) and condensed trigonal prisms around As atoms, which form a “shamrock” trefoil with a Th atom in the center (bottom panel).

**Figure 2:**
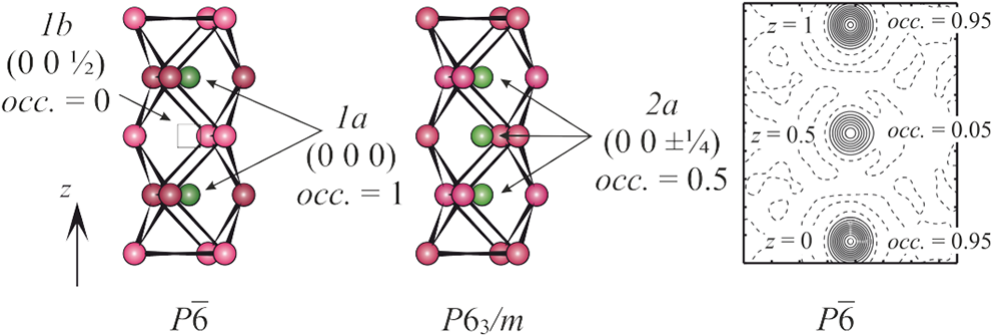
Atomic decoration around the [001] line in two models of the Th_2_Fe_12_As_7_ crystal structure (left and middle panels) and the distribution of the experimental difference electron density along the [001] line ((010) plane) (right panel). Iron atoms are magenta, whereas arsenic atoms are green. Isolines are drawn with a step of 1 e/A^–3^. Zero level is shown as a dashed lines.

**Figure 3:**
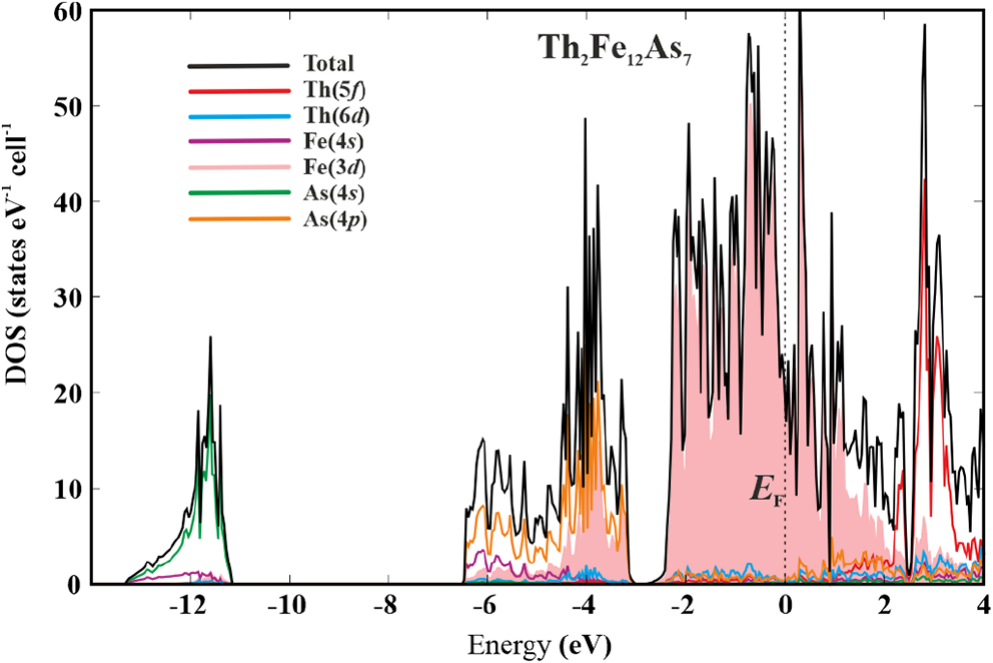
Electronic density of states (DOS) for Th_2_Fe_12_As_7_ together with the contributions of the essential atomic states.

**Figure 4:**
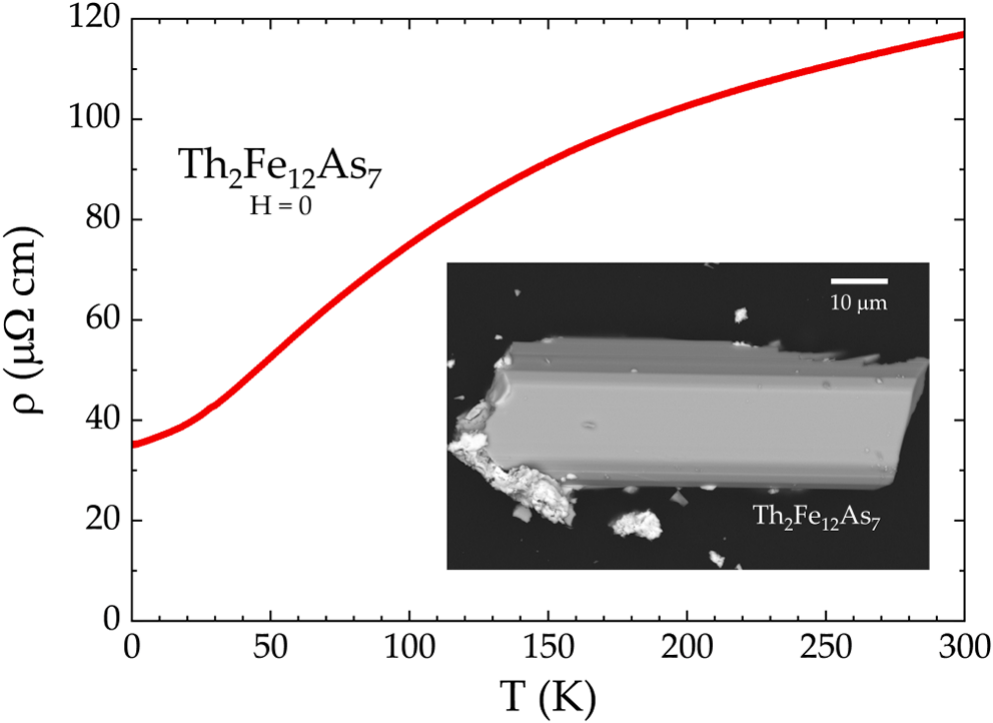
Electrical resistivity of Th_2_Fe_12_As_7_ in *H* = 0. Inset: Single crystals of Th_2_Fe_12_As_7_ have needle-like morphology with the long axis being the [001] direction.

While in the *P* 6 model, the position at *z* = 0 is occupied and one at *z* = *^1^/_2_* is empty, in the *P*6_3_/*m* model, both positions are described by one 2-fold crystallographic site (the lowest multiplicity allowed in this space group) with an occupancy factor of 0.5.

Experimentally, both models differ in the extinction conditions for the 00*l* reflections: in the first one, no extinctions should be observed, while in the second one, only reflections with even *l* should be present. Among the 6283 reflections measured for the Th_2_Fe_12_As_7_ crystal, only four such reflections were observed due to the small lattice parameter *c* = 3.8645(2) Å—one 004 with strong intensity (*F* ^2^/σ*F* ^2^ ≈ 428/11) and three very weak ones (001, 003, and 005 with *F* ^2^/σ*F* ^2^ of ca. 4.2/0.3, 1.1/0.3, and 0.6/0.3, respectively). Such insufficient statistics do not allow one to exclude one of the space groups *a priori*. Therefore, the comparison has to be done by full structure determination and refinement.

The crystal structures in both groups were solved by the charge-flip technique within the WinCSD program package.^23^ The refinement of the model, being well in agreement with the structure type Zr_2_Fe_12_P_7_,^37^ in the space group *P* 6 converged quickly with the residual values of *R_F_* = 0.0446 for 6174 observed reflections and *R_F_* = 0.0412 for 1412 symmetry independent reflections. The structure solution in the *P*6_3_/*m* space group required the split of one of the Fe positions and half-occupation of the As positions on the [001] axis, but nevertheless revealed the essentially larger residual values of *R_F_* = 0.0776 for 5727 observed reflections and *R_F_* = 0.0778 for 728 symmetry independent reflections. It is, therefore, clear that the *P* 6 space group describes the crystal structure of Th_2_Fe_12_As_7_ better. The noncentrosymmetric group raises the question regarding the presence of inversion twinning in the investigated crystal. Employment of the inversion matrix for the description of twinning in the calculation of reflection intensity reduces the residual to *R_F_* = 0.0342 for 5740 observed reflections. Therefore, the reduction of the residual may have other reasons. The difference density map calculated without any atoms on the [001] line (Figure 2, right) reveals, in addition to the strong maximum at the origin, a weaker maximum at (00*^1^/_2_*). Further refinement of the occupation parameters of these positions (restricted in sum to 1) yields the occupation ratio of 0.95/0.05. Partial occupation of the (00*^1^/_2_*) position can be understood as a presence of equidistant chains of As atoms along [001], with the origins shifted by (00*^1^/_2_*) in the structure. This finding indicates an intermediate character of the real structure of Th_2_Fe_12_As_7_ situated between the two models described in space groups *P* 6 and *P*6_3_/*m*.

The calculated density of states (DOS) for Th_2_Fe_12_As_7_ is shown in Figure 3. The non-zero value of DOS at the Fermi level is in accordance with metallic behavior observed in the measurement of electrical resistivity—see Figure 4. The value of the residual resistivity ratio (RRR) is 3, which is fairly low and can possibly be attributed to twinning. At low temperatures, the behavior does not follow the ρ ∝ *T* ^2^ behavior expected for a Fermi liquid.

The value of the DOS at the Fermi level *E_F_* ≈ 20 eV allows us to estimate the value of the Sommerfeld coefficient γ = 50 mJ mol_F.U._^–1^ K^–2^. Given the lack of specific heat data, it is currently not possible to compare this estimate with an experimental value. The DOS shows three characteristic regions below the Fermi level (Figure 3). The low-energy range (−13.5 < *E* < −11 eV) is contributed mainly by the s states of As. This would hint toward As lone pair formation. However, the essential contribution of the s states of Fe in this region indicates the bonding character of these electrons, see the bonding analysis below. The middle-energy part of DOS (−7 < *E* < −3 eV) can be virtually split into two subregions. Below −5 eV, the p states of As and s states of Fe make essential contributions, above −5 eV, p states of As play the main role together with the *d* states of Fe. The third range (−3 eV < *E* < *E_F_*) is mainly formed by the d states of Fe. The d and f states of Th contribute homogeneously to all three DOS regions below the Fermi level, indicating the essential level of charge transfer from Th to the Fe–As substructure. The Fermi level is located close to a deep dip (pseudo gap) in the total DOS, which is often observed in intermetallic compounds with a high level of covalency in the bonding. This is the case for Be–Ru compounds,^38^ Mg_3–*x*_Ga_1+*x*_Ir,^39^ and Hf_2_B_2–2δ_Ir_5+δ_.^40^

The crystal structures of the compounds assigned to the Zr_2_Fe_12_P_7_ (*TM*1_2_ *TM*2_12_ *E*_7_) structure type or its noncentrosymmetric variety Cl_2_F_12_Ba_7_ (see above) are usually described in the literature as a pattern constituted of two types of edge-condensed columns of triangular prisms [*TM*1_2_ *TM*2_4_] and [*TM*2_6_] with the transition metals at the vertices and the *E* element in the middle. Through the shifting of neighboring columns by *^c^/_2_*, the triangular prisms are side-capped, and the crystallographic coordination number of As is nine (see Figure 1).

**Figure 5:**
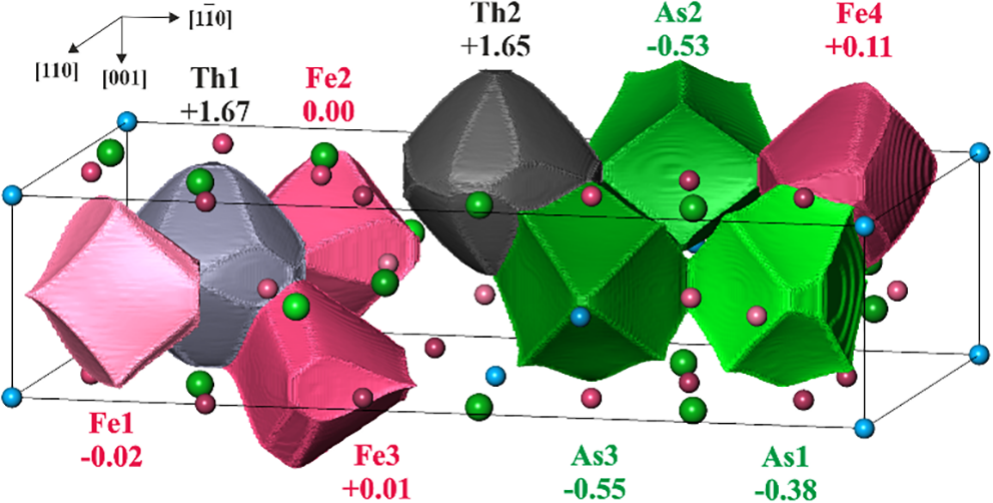
QTAIM atomic shapes and effective charges in Th_2_Fe_12_As_7_. Black lines show the borders of the calculated region (orthohexagonal unit cell).

**Figure 6:**
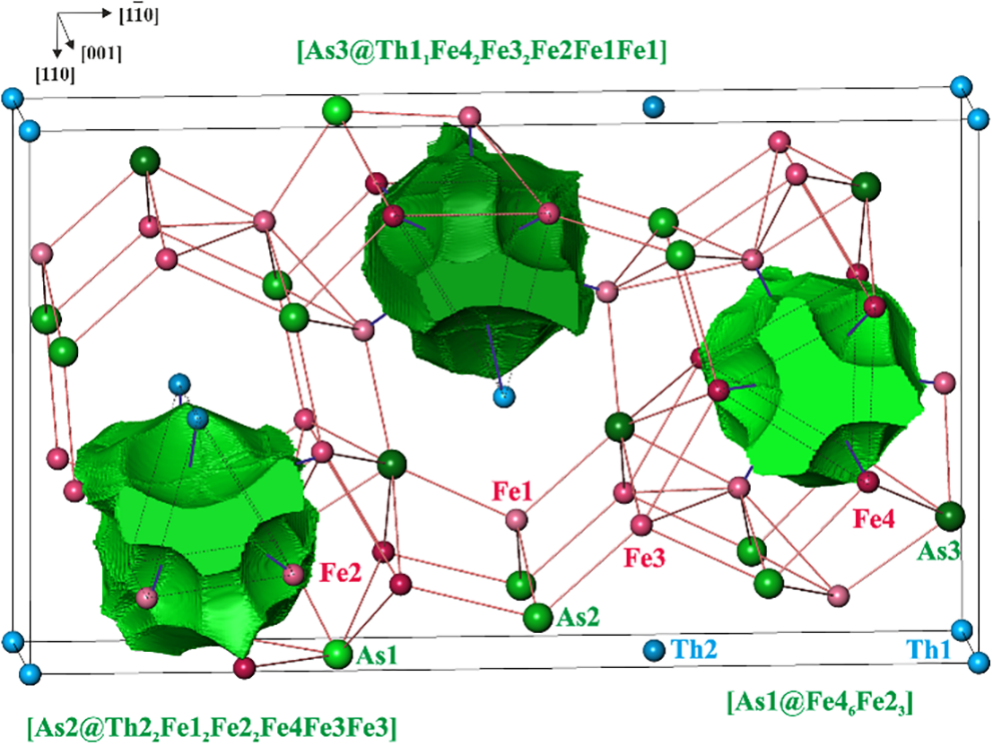
Superbasins of the As anions in Th_2_Fe_12_As_7_. Red lines represent the shortest interatomic contacts within the Fe–As framework, blue lines show the distances within the coordination sphere of each As species, the black lines show the borders of the calculated region (orthohexagonal unit cell), and the coordination spheres for each As atom are also given.

**Figure 7:**
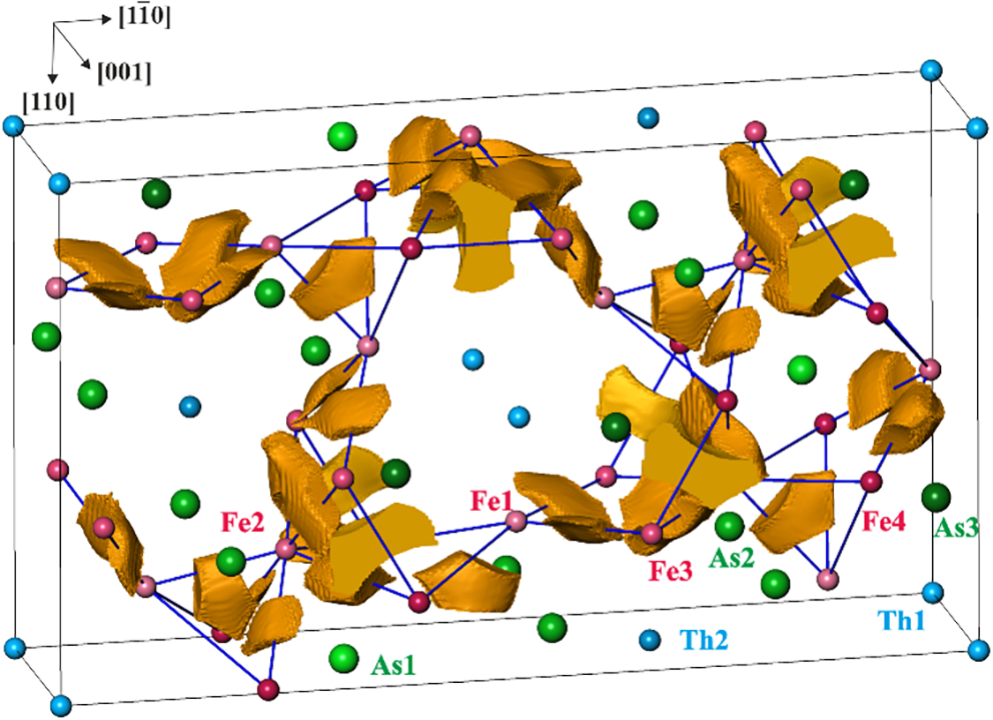
Bond basins of the 2a-Fe–Fe bonds in Th_2_Fe_12_As_7_. Blue lines are the shortest interatomic contacts, and black lines show the borders of the calculated region (orthohexagonal unit cell).

The bonding confirmation for this crystallographic description was performed by analysis of atomic interactions using quantum chemical techniques in positions space. Zero-flux surfaces in the gradient vector field of electron density in Th_2_Fe_12_As_7_ form the boundaries of electron density basins, which represent atomic regions according to the Quantum Theory of Atoms in Molecules (QTAIM,^32^ Figure 5). The Th QTAIM atoms (Figure 6) show rather spherical shapes, typical for cations in the QTAIM representation—see, for example, Sr in Sr_8_Si_46_ ^41^ or Y in Y_4_Be_33_Pt_16_.^42^ More plane faces of the atomic shapes of Fe and As indicate lower polarity (increased covalency) of the interaction in the given directions and appear if the electronegativity difference between the bond partners is smaller.^43^ The As species in Th_2_Fe_12_As_7_ show shapes with concave faces toward the Th and Fe ligands, well in agreement with the charge transfer.

Integration of the electron density in spatial atomic regions, defined in QTAIM, yields their electronic populations. The subtraction of latter from the respective atomic numbers results in the QTAIM effective atomic charges, shown in Figure 5. Both Th species in Th_2_Fe_12_As_7_ carry a relatively large positive charge, clearly revealing their cationic role in this compound. It is worth mentioning that the charge values of Th are essentially larger than the observed values for alkaline earth cations (between +1.11 and +1.54),^41^ or even those for rare-earth cations in Y–Ga compounds (<+1.5)^44^ and Pr in *hp*-Pr_2_Si_7_ (+1.10 or +1.20).^45^ According to the electronegativity differences, As species show negative effective charges between −0.38 and −0.55. The most interesting are the effective charges of the Fe species, which vary around zero (−0.02 to +0.11). The Bader charges agree with the strong electron transfer from Th cations to the Fe–As substructure (see the electronic DOS above) and indicate polar covalent bonding between Fe and As. Further details of atomic interactions were obtained from the combined analysis of electron density and electron-localizability indicators—in its ELI-D representation^46^—according to the strategy described in ref 29.

The spherical distribution of the ELI-D characteristic observed in noninteracting atoms is disrupted by the formation of chemical bonds (Figure 7). Attractors (maxima) may appear in the regions of valence or penultimate shells, signaling bonding and revealing its geometrical location.^29^ In the case of Th_2_Fe_12_As_7_, the penultimate shells of As species are still spherical—see Figure S1—which agrees with the dominant role of the s and p electrons in the electronic DOS. The ELI-D maxima appear in the valence region, as shown in Figure S1. Each of these attractors (maxima) has its own ELI-D basin, which is determined, like the QTAIM atomic basins, by the zero-flux surfaces in the gradient vector field of ELI-D, see Figure S2. The number of common surfaces of a bonding basin with the attached core (penultimate shell) basins defines the atomicity of the bonding basin and characterizes the number of atomic species participating in this bond. The common surfaces with the attached core basins are pronouncedly concave, as is evident from Figure 5. What is characteristic of Th_2_Fe_12_As_7_ is that most basins with the participation of As represent four or even six atomic bonds. Arsenic contributes the larger part of the bond basin populations, i.e., all these bonds have moderate polarity between 0.21 and 0.45 on the scale between 0 (covalent nonpolar bond) and 1 (ionic bond close-shell situation).^47^

Combining all bond basins with participation of the As species yields superbasins, characterizing the As anions. The populations of the superbasins are 8.58, 9.31, and 9.19 electrons for As1, As2, and As3, respectively. The larger values for As2 and As3 are caused by Th species in their environment, while As1 has only Fe atoms as ligands. All three superbasins are nine-atomic, which means they share common surfaces with nine atomic core basins (Figure 6). Thus, the topology of the superbasins resembles the triangular prisms, which were deduced from the analysis of the interatomic distances described above, and therefore supports the crystallographic interpretation, see Figure 8.

Interestingly, the bonding analysis of Th_2_Fe_12_As_7_ shows an additional new bonding feature. Besides the multiatomic bond basins, the electron-localizability approach reveals two-atom bond basins with low bond populations between the Fe species, as presented in Figure S2. They create a three-dimensional network of interconnected Fe atoms; see Figure 7.

**Figure 8:**
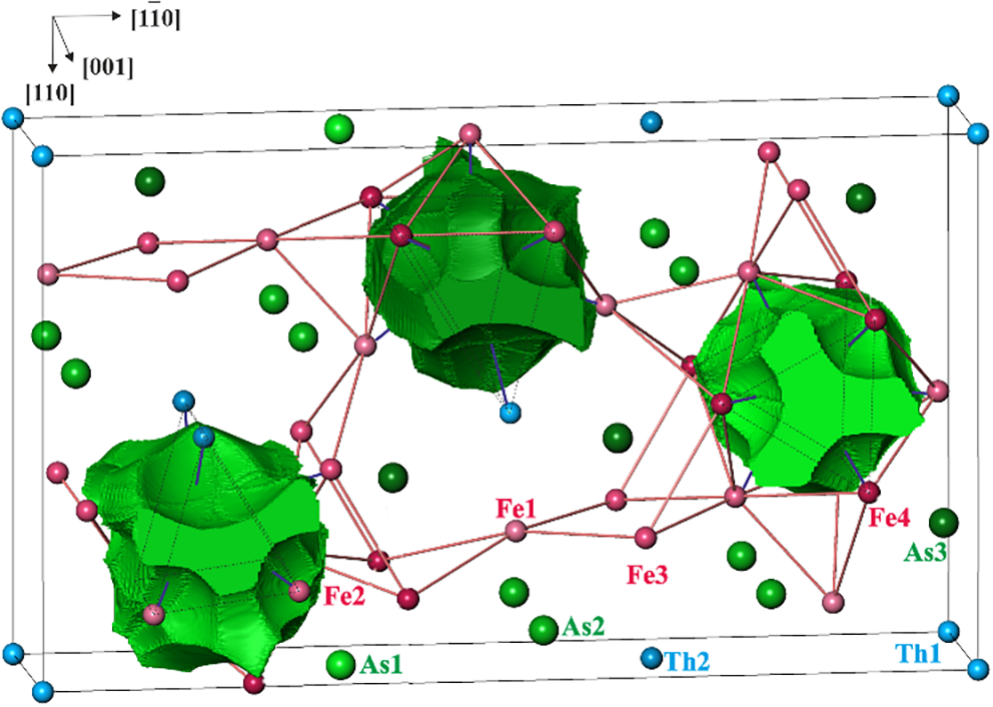
Bonding picture in Th_2_Fe_12_As_7_: As anions, visualized by the bond superbasins (green), interacting ionically with the cationic Th species (blue), mediated by the neutral three-dimensional Fe framework (red lines).

## IV Summary and Conclusions

In summary, the bonding picture of Th_2_Fe_12_As_7_, shown in Figure 8, resembles the crystallographic one, supporting the essential role of triangular prisms around As atoms found from the analysis of interatomic distances. From the bonding point of view, the prisms are geometric places of anionic components of the structure. The cationic components of the structure are represented by the Th species. A new feature—revealed by the bonding analysis—is the three-dimensional framework of two-atomic bonds between the Fe atoms, which are essentially neutral (in the sense of effective charges). It plays the role of a bonding mediator within the [Fe–As] framework. The bonding is multiatomic polar in the whole structure, besides the Fe–Fe interactions. This shows that the traditional interpretation of such compounds as anionic frameworks with embedded cations is only the starting point of the bonding description. Th_2_Fe_12_As_7_ is likely a paramagnet and displays metallic electrical resistivity, as supported by a non-zero density of states at the Fermi level. However, it is possible that this compound exhibits magnetic ordering below 0.4 K, a scenario that may arise from frustration among iron atoms arranged in a triangular lattice. The synthesis of large single-phase specimens is therefore of particular interest for future studies.

## Supplementary Material



## Notes

ELI-D maps, together with additional crystallographic data (PDF)
