# Supplementary material for: Thorium-Based Iron Arsenide with Fe–Fe Bonding
Source: Inorg Chem. 2025 Oct 8;64(41):20577–83. doi: 10.1021/acs.inorgchem.5c02207 (PMC12541701; doi:10.1021/acs.inorgchem.5c02207)
Supplement: Supplementary file 1 [file ic5c02207_si_001.pdf]

Supporting Information

A thorium-based iron arsenide with Fe–Fe bonding

Nazar Zaremba,[1] Mitja Krnel,[1] Konstantin Semeniuk,[1] Yurii Prots,[1] Lev Akselrud,[2]
Ulrich Burkhardt,[1] Andreas Leithe-Jasper,[1] Eteri Svanidze,[1,*] and Yuri Grin[1]

[1]*Max Planck Institute for Chemical Physics of Solids, 01187 Dresden, Germany*

[2]*Department of Inorganic Chemistry, Ivan Franko National University of Lviv, Lviv 79000, Ukraine*

[*]*Eteri.Svanidze@cpfs.mpg.de*

TABLE III. Atomic coordinates and equivalent displacement parameters (in Å^2) of $Th_2Fe_{12}As_7$ refined in $P6_3/m$ space group. [a]

Atom	Wyckoff site	x/a	y/b	z/c	U_{eq} Å^2
Th1	2*c*	⅔	⅓	¾	0.0098(3)
Fe1	6*h*	0.6213(4)	0.0506(3)	¼	0.0111(8)
Fe2	6*h*	0.1012(8)	0.2147(8)	¼	0.013(2)
Fe3	6*h*	0.1228(7)	0.2761(8)	¼	0.011(2)
As1	6*h*	0.4058(2)	0.1121(2)	¼	0.0093(5)
As2	2*a*	0	0	¼	0.0088(11)

[a] Occupancy for Fe2 and Fe3 are 0.477(9) and 0.523(9) respectively. As2 is half occupied.

TABLE IV. Interatomic distances in $Th_2Fe_{12}As_7$ refined in $P\bar{6}$ space group. [a]

Atoms		δ, Å	Atoms		δ, Å	Atoms		δ, Å
Th1–	6As3	3.017(1)	Fe3–	1As2	2.414(3)	As1–	2As4	*1.9322(2)*
	6Fe1	3.144(2)		2As3	2.422(2)		3Fe2	2.313(2)
	3Fe3	3.306(2)		1As2	2.459(3)		6Fe4	2.621(2)
	3Fe5	3.310(5)		1Fe5	2.540(5)		6Fe5	2.936(4)
	3Fe4	3.745(3)		2Fe2	2.699(2)	As2–	1Fe5	2.380(5)
	2Th1	3.8645(3)		1Fe4	2.713(4)		1Fe3	2.414(3)
	3As2	3.932(2)		2Fe1	2.808(3)		2Fe1	2.417(2)
Th2–	6As2	3.040(2)		2Th2	3.174(2)		2Fe2	2.419(2)
	6Fe3	3.174(2)		1Th1	3.306(2)		1Fe3	2.459(3)
	3Fe2	3.209(2)	Fe4–	1As4	*1.771(3)*		1Fe4	2.476(3)
	3Fe1	3.338(2)		1As2	2.476(3)		2Th2	3.040(2)
	2Th2	3.8645(3)		2As1	2.621(2)	As3–	1Fe5	2.380(5)
	3As2	3.978(2)		2As3	2.660(2)		1Fe2	2.349(3)
Fe1–	1As3	2.390(3)		1Fe3	2.713(4)		1Fe1	2.390(3)
	1As3	2.416(3)		2Fe2	2.822(3)		1Fe1	2.416(3)
	2As2	2.417(2)		2Fe2	2.878(3)		2Fe3	2.422(2)
	1Fe2	2.588(4)		2Fe1	3.022(3)		2Fe5	2.430(3)
	2Fe5	2.780(4)		2Fe4	3.068(4)		2Fe4	2.660(2)
	2Fe3	2.808(3)	Fe5–	1As4	2.210(5)		2Th1	3.017(1)
	2Fe4	3.022(3)		1As2	2.380(5)	As4–	3Fe4	*1.771(3)*
	2Th1	3.144(2)		2As3	2.430(3)		2As1	*1.9322(2)*
	1Th2	3.338(3)		1Fe3	2.537(5)		3Fe5	2.210(5)
Fe2–	1As1	2.313(2)		2Fe1	2.776(4)		6Fe2	3.014(2)
	1As3	2.349(3)		2As1	2.936(4)			
	2As2	2.419(2)		2Fe2	2.936(4)			
	1Fe1	2.588(4)		2Fe2	3.010(4)			
	2Fe3	2.699(2)						
	2Fe4	2.822(3)						
	2Fe4	2.878(3)						
	2Fe5	2.940(4)						
	2Fe5	3.010(4)						
	2As4	3.014(3)						
	1Th2	3.209(2)						

[a] The distances shown by italics indicate only distances between partially occupied or splitted atoms, but do not reflect real contacts in the structure.

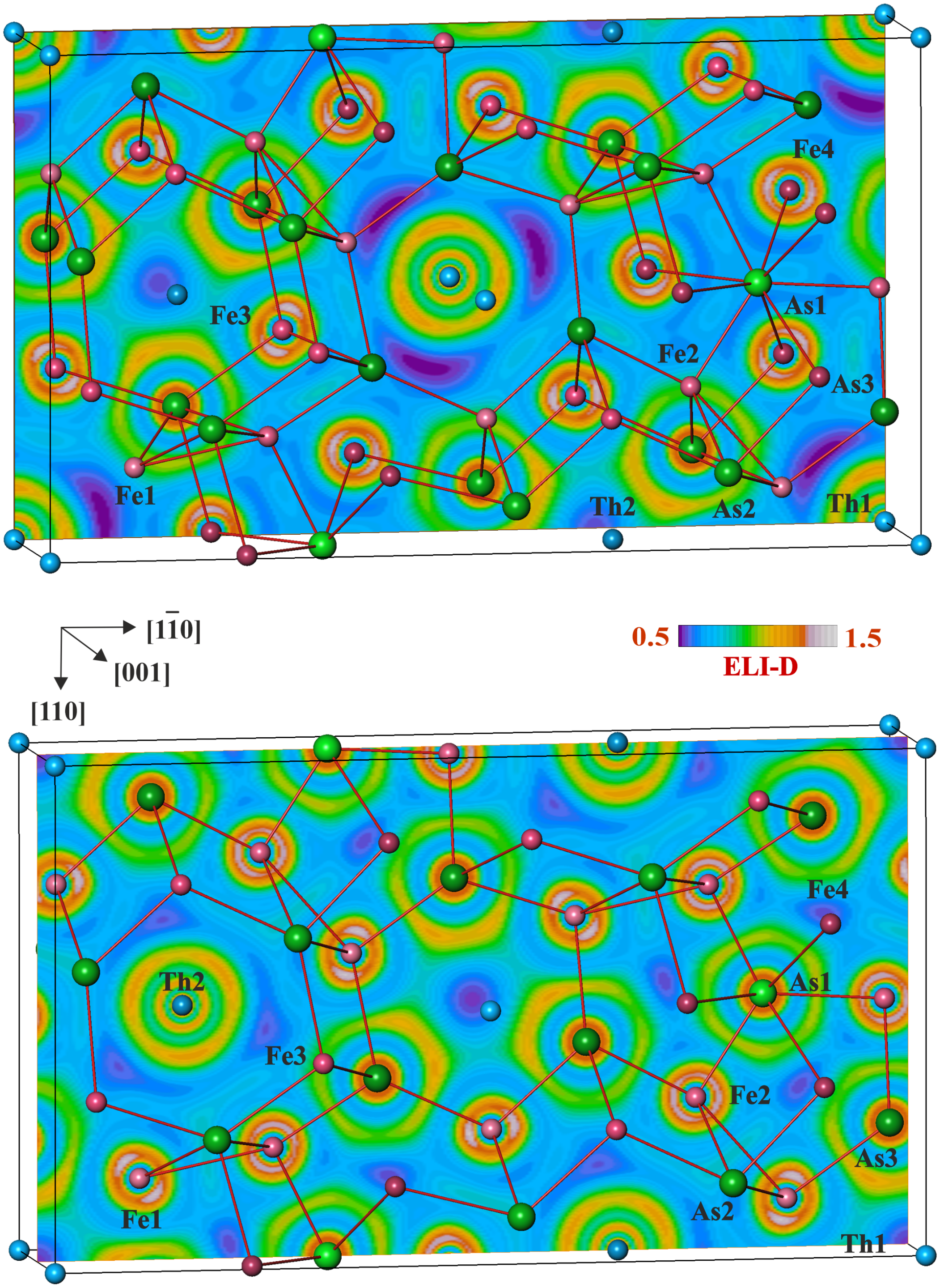

FIG. 9. Distribution of ELI-D in $Th_2Fe_{12}As_7$ at $z = 0$ (top) and $z = 0.5$ (bottom).

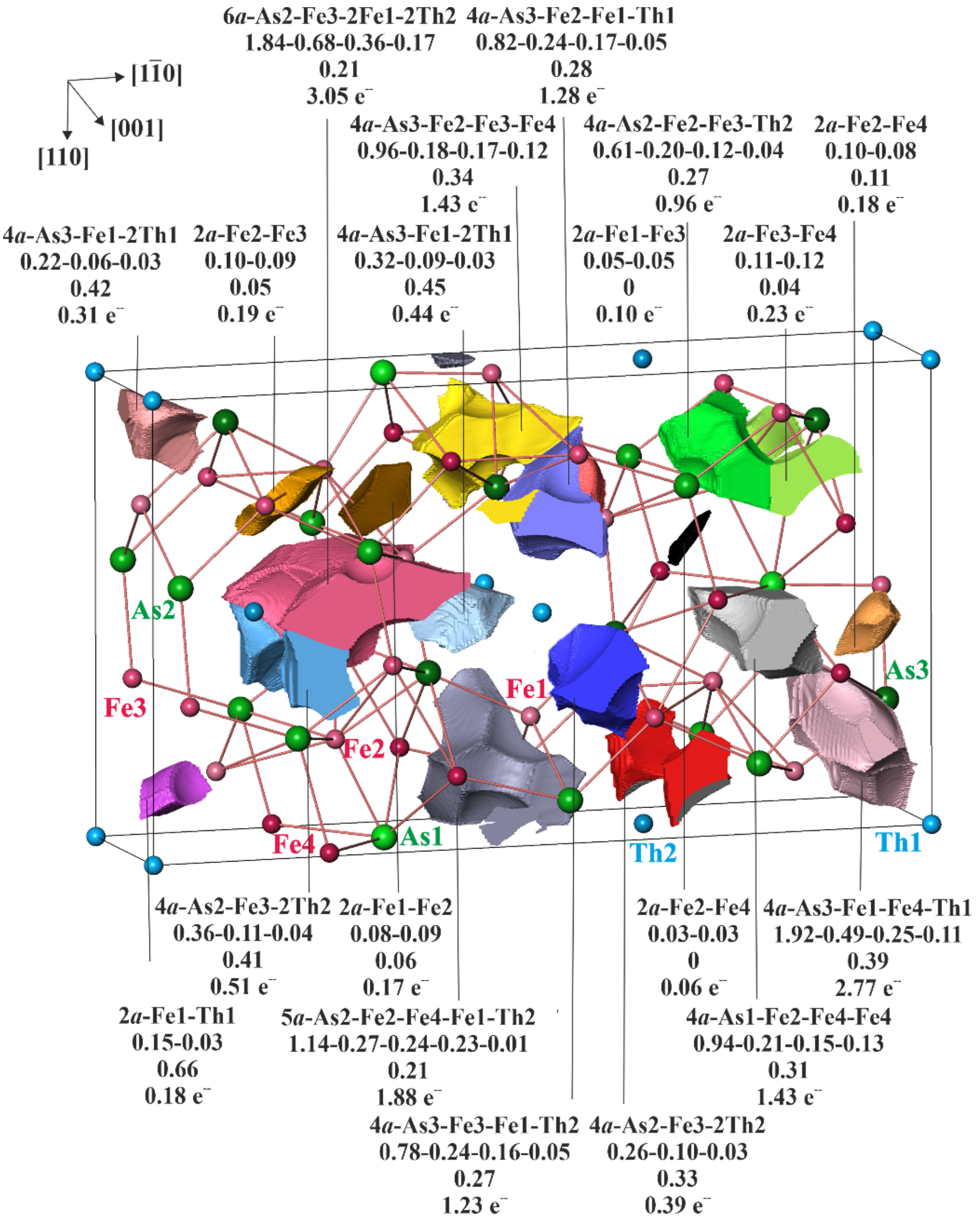

FIG. 10. ELI-D bond basins in $Th_2Fe_{12}As_7$. For each basin is given its atomicity and list of contribution atoms (first line), contributions of each atom to the bond population (second line), bond polarity (polar character, third line) and total bond basin population (fourth line). Red lines – the shortest interatomic contacts, black lines show the borders of the calculated region (ortho-hexagonal unit cell).

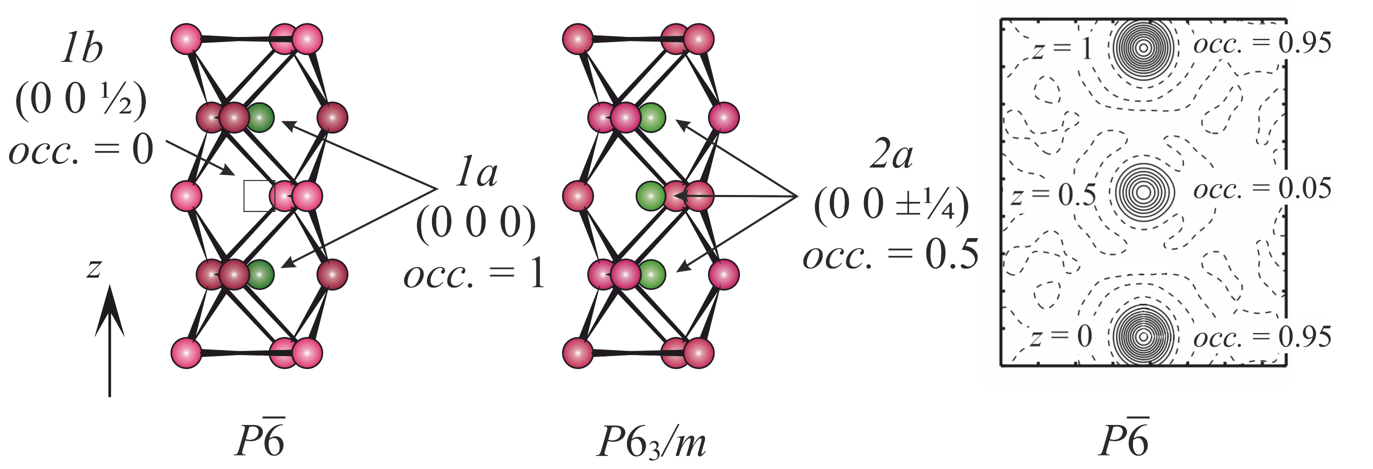

FIG. For Table of Contents Only. The representation of atomic distribution around the [001] line for the two models of the $Th_2Fe_{12}As_7$ crystal structure (left and middle). The distribution of the experimental difference electron density along the [001] axis for $Th_2Fe_{12}As_7$ (right).
